# Flexible Meandered UHF RFID Tag Antenna on a Paper-Backed Substrate: Impact of Chip Placement and Material Proximity for Industrial Applications

**DOI:** 10.3390/s26092598

**Published:** 2026-04-23

**Authors:** Hamza Othmani, Jamel Smida, Mohamed Karim Azizi

**Affiliations:** 1 Microwave Electronics Research Laboratory, Faculty of Sciences of Tunis, University of Tunis El Manar, Tunis 2092, Tunisia; jsmida@um.edu.sa (J.S.); mohamedkarim.azizi@isamm.uma.tn (M.K.A.); 2 Department of Industrial Engineering, College of Applied Science, AlMaarefa University, Riyadh 11597, Saudi Arabia; 3 Higher Institute of Arts and Multimedia, University of Manouba, Manouba 2010, Tunisia

**Keywords:** UHF RFID, tag antenna, meandered dipole, chip placement, miniaturization, material proximity, industrial applications

## Abstract

In this work, the design and experimental validation of passive UHF RFID tag antennas are presented with the objective of evaluating the impact of chip placement and miniaturization approaches on tag performance. Four initial antenna layouts were developed by varying the position of the RFID integrated circuit within a coupling loop. The results show that chip placement directly affects the coupling-loop efficiency, the antenna–chip matching condition, and the practical tolerance of the structure to fabrication-related variations. Simulations and measurements identified Antenna 1 as the best-performing reference configuration, exhibiting the most favorable impedance behavior around 866 MHz and a measured power sensitivity of −16.3 dBm. Based on this reference design, a miniaturized version (Antenna 5) was obtained by integrating meander lines and capacitive end-loading, reducing the physical size while maintaining resonance at 866 MHz. Both structures were fabricated and evaluated using a Voyantic Tagformance measurement system, with read-range measurements performed under free-space conditions and in proximity to dielectric and conductive materials. The results demonstrate a maximum read range of 8.6 m for Antenna 1 in free space, while Antenna 5 preserved a read range of 6.3 m. In the presence of copper, Antenna 1 maintained a read range of 3 m, whereas Antenna 5 achieved approximately 0.5 m, highlighting the trade-off between miniaturization and robustness under conductive loading.

## 1. Introduction

Radio Frequency Identification (RFID) has become a key enabling technology in modern information and communication systems, supporting a wide spectrum of applications that require efficient, reliable, and contactless identification. In supply chain management [1], RFID enables real-time traceability [2], and inventory optimization [3], while in healthcare it enhances patient safety [4,5], pharmaceutical monitoring [6], and medical equipment tracking [7,8]. In industrial automation, RFID contributes to intelligent production flows, asset monitoring [9], and predictive maintenance strategies [10]. Moreover, RFID-based access control systems ensure secure authentication in transportation networks [11], and service infrastructures [12]. Beyond these application-specific contexts, RFID plays a central role in digital transformation frameworks by enabling robust object identification [13]. RFID technology influences data integration within cyber–physical systems [14].

An RFID system typically consists of a reader equipped with a dedicated antenna and a tag integrating both an antenna and an integrated circuit (IC). Depending on their powering mechanism, RFID tags are classified as passive, semi-passive, or active. Passive tags operate exclusively by harvesting energy from the incident electromagnetic field, semi-passive tags incorporate a small battery to support internal circuitry, and active tags rely on an internal power source for communication and sensing functionalities [15]. RFID technology spans several frequency bands, including Low Frequency (LF, 125–134 kHz), High Frequency (HF, 13.56 MHz), and Ultra-High Frequency (UHF, 860–960 MHz), which supports long-range identification and high data-rate operation [16]. Among these, the UHF band has emerged as the dominant choice for large-scale logistics and industrial deployments due to its extended read range, rapid inventory capability [17], and compatibility with global supply-chain standards [18].

Despite its widespread adoption, the design of UHF RFID tag antennas continues to face several unresolved challenges. One of the most critical issues is achieving reliable conjugate impedance matching between the antenna and the highly capacitive RFID IC [19], as any mismatch directly degrades power transfer efficiency and limits the achievable read range [20,21]. In addition, antenna sensitivity remains a limiting factor, particularly in industrial environments where tags are exposed to diverse surrounding materials that significantly alter their electromagnetic behavior [22]. Proximity to dielectric or metallic objects often results in resonance detuning, bandwidth reduction [23], and severe radiation efficiency degradation [24]. Environmental conditions such as humidity, temperature variations [25], and mechanical stress further complicate the operation of the tag and impose additional constraints on long-term reliability and robustness [26].

Another major design challenge lies in antenna miniaturization. Compact tag antennas are highly desirable for integration into modern packaging [27], wearable platforms [28], and embedded sensing systems [29,30]. However, reducing the antenna footprint typically leads to increased losses, narrower bandwidth, and greater sensitivity to material loading effects [31]. To address these issues, various miniaturization strategies have been explored, including meander-line geometries [32], folded dipole structures [33], and capacitive loading techniques [34]. While these approaches enable size reduction, they often introduce trade-offs between efficiency, bandwidth, robustness, and manufacturability, particularly when flexible or unconventional substrates are considered [35].

In this context, the development of flexible UHF RFID tag antennas capable of maintaining stable performance under material proximity and industrial constraints remains an open research problem. Substrate selection plays a crucial role in this regard, as unconventional and low-cost materials may offer advantages in terms of flexibility and sustainability but may also introduce additional electromagnetic uncertainties. Furthermore, chip placement tolerance and robustness against environmental loading are rarely addressed jointly in existing studies [36], despite their practical relevance for large-scale industrial deployments [37]. Recent studies have also highlighted the growing relevance of RFID within sustainability-oriented frameworks, including circular-economy deployment and greener electronic manufacturing. In particular, RFID has been identified as an enabling technology for traceability and resource-circulation strategies in circular systems, while printed and substrate-engineered RFID platforms are increasingly investigated in the context of more sustainable electronics and eco-friendly manufacturing routes [38,39].

This paper presents the design of a flexible meandered UHF RFID tag antenna fabricated on a paper-backed substrate, with a particular focus on robustness and practical deployment constraints. An experimental investigation is conducted to evaluate the tolerance of the antenna to variations in RFID chip placement and to analyze the impact of such variations on impedance matching and read-range stability. The robustness of the proposed design is further examined under proximity to industrially relevant materials, including conductive surfaces. It should be noted, however, that the proposed structure is not intended as a dedicated on-metal RFID tag for direct attachment to metallic objects, but rather as a flexible tag whose performance is evaluated under nearby material-loading conditions. The performance of the proposed antenna is comparatively assessed against state-of-the-art UHF RFID tag antennas in terms of physical size, operational bandwidth, and achievable read range. In addition, an experimental benchmark against two commercially available UHF RFID tags is performed under identical free-space and material-proximity conditions, providing a realistic reference for evaluating read-range degradation trends and overall robustness in representative industrial scenarios.

The novelty of this work does not reside in proposing a completely new meandered UHF RFID tag topology. Rather, it lies in the systematic comparative analysis of four chip-placement and coupling-loop configurations implemented on a flexible paper-backed substrate, in order to identify the most favorable matching condition for passive UHF RFID operation. Based on this comparative study, a miniaturized structure is then derived and experimentally evaluated under free-space and material-proximity conditions. In addition, the proposed designs are benchmarked against commercial RFID tags under identical measurement conditions, providing a practical assessment of robustness and performance in representative industrial scenarios.

The remainder of this paper is organized as follows. Section 2 introduces the theoretical background and equivalent circuit modeling. Section 3 presents the antenna configurations and experimental validation. The measured and simulated results are then discussed, followed by concluding remarks.

## 2. Antenna Design and Theoretical Background

### 2.1. Antenna Geometry and Operating Band

**Figure 1:**
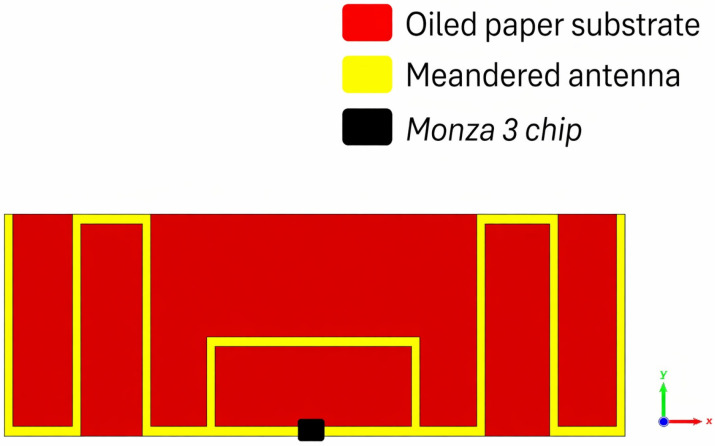
Proposed meandered UHF RFID tag design.

The proposed UHF RFID tag prototypes were fabricated from a flexible adhesive copper foil supported by a paper-based backing, with an overall thickness of approximately 0.2 mm. This material is routinely used in our laboratory for rapid RFID tag prototyping using a Summa F1625 cutting machine(Summa nv (Summa), Gistel, Belgium), mainly because it provides a practical, flexible support for the thin copper layer and enables straightforward fabrication of the tag geometry. The material used in this work did not provide certified dielectric parameters of the backing layer, such as frequency-dependent relative permittivity or dielectric loss tangent. Therefore, the substrate was not treated here as a fully characterized dielectric material. In particular, the value $\varepsilon_{eff}=3.87$ was not experimentally determined for this material at a specific frequency. Instead, it was adopted only as an effective design parameter during the numerical stage in order to enable preliminary tuning of the antenna response around the target UHF RFID operating frequency of 866 MHz. Because the paper-based backing is very thin (about 0.2 mm), its dielectric contribution was treated here as a secondary effect compared with the dominant influence of the conductor geometry and the antenna–chip tuning process. The final antenna dimensions were then obtained through iterative electromagnetic optimization and prototype tuning under identical fabrication conditions. This approach ensures that the final antenna performance is primarily validated through experimental measurements on the fabricated prototypes rather than through precise dielectric modeling of the substrate alone. In addition, no direct loss-tangent measurement was carried out, since such a characterization was not feasible with the measurement setup available to the authors for a backing layer of such small thickness, whereas the available material characterization approach requires significantly thicker samples. Therefore, no reliable loss tangent data were available for the material used, and the reported results should be interpreted as a comparative prototype study of the proposed antenna configurations rather than as a rigorous dielectric characterization of the substrate. In addition, the exact composition and long-term environmental stability of the paper-based backing were not systematically investigated in this study. From an application point of view, this implies that possible variations under humidity and temperature fluctuations should be addressed in future work before fully validated industrial deployment. The radiating element is made of copper with a thickness of 0.035 mm. The design operates within the UHF RFID band of 865–868 MHz, with a nominal resonance at 866.6 MHz. The initial layout is derived from a half-wavelength dipole optimized for the UHF range and is shortened through meander sections to reduce the physical length while maintaining the required electrical path. Figure 1 shows the overall geometry of the proposed meandered UHF RFID tag.

In this study, four reference configurations were realized, differing only in the placement of the IC along the dipole loop region, in order to provide a systematic evaluation of chip-location effects on input impedance and matching.

### 2.2. Electrical Model of the RFID Tag

**Figure 2:**
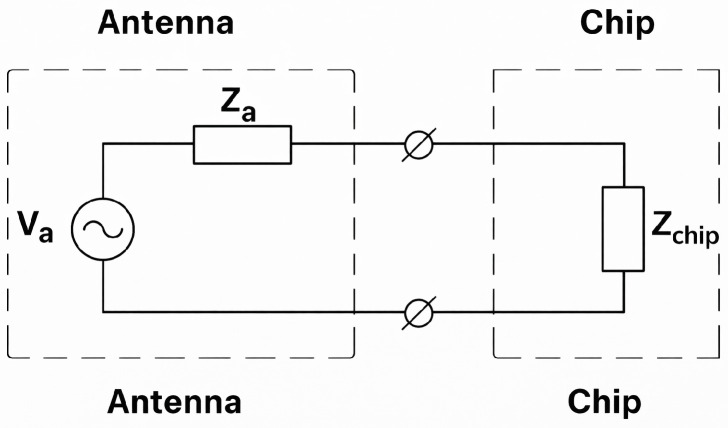
Equivalent circuit representation of the RFID tag.

The equivalent circuit of an RFID tag is shown in Figure 2. The source $V_{a}$ corresponds to the open-circuit RF voltage at the antenna terminals. The impedance of the chip reflects both its input properties and the parasitic elements of the package. Both $Z_{a}$ and $Z_{chip}$ vary with frequency, and $Z_{chip}$ may also depend on the incident power level. For proper operation, the antenna is designed to achieve conjugate matching with the chip at the minimum activation power.

The antenna is modeled by its complex impedance

(1) $$Z_{a}=R_{a}+jX_{a}$$

**Figure 3:**
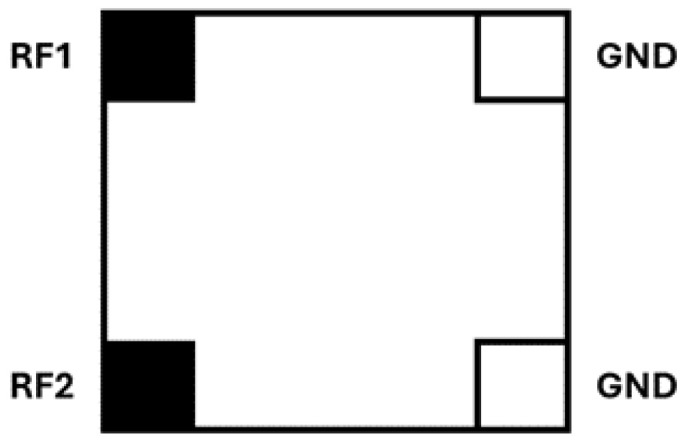
Pad configuration of the Impinj Monza 3 chip (RF1, RF2, GND, GND), redrawn from the manufacturer documentation.

In this work, the Impinj Monza 3 RFID chip was selected as the integrated circuit of the tag. Although this chip is not among the most recent generations of UHF RFID chips, it remains suitable for laboratory prototyping because of its practical pad configuration. As illustrated in Figure 3, the chip provides four external pads, namely RF1, RF2, GND, and GND, which facilitates manual alignment and electrical connection during the fabrication of the tag prototypes. The chip was not interfaced as a bare die through a custom fixture kit. Instead, the prototypes were realized using the practical four-pad chip configuration (RF1, RF2, GND, GND), which enabled direct manual soldered connection to the antenna structure. In the revised manuscript, it is also clarified that the impedance and sensitivity values reported in the manufacturer documentation refer to the chip configuration specified in the datasheet. The use of a more recent RFID chip with higher sensitivity could potentially improve the read-range performance. However, because modern chips generally exhibit different complex impedance and package-dependent parasitic effects, such an upgrade would require rematching or partial redesign of the antenna. Therefore, practical attachment and soldering may introduce additional parasitic effects and may slightly modify the effective impedance seen in the fabricated prototype.

The chip is represented by

(2) $$Z_{chip}=R_{chip}-jX_{chip}$$

The design objective is to satisfy the conjugate-matching condition at the nominal frequency $f_{0}$

(3) $$Z_{a}=Z_{chip}^{*}$$

which maximizes the power delivered to the chip and represents the standard design criterion in the development of UHF RFID tags.

### 2.3. Electrical Modeling of the RFID Chip

The RFID integrated circuit (IC) is a fundamental component in the operation of the UHF tag, as it defines the conditions for energy transfer from the reader and backscatter data communication. In this study, the Impinj Monza 3 IC is selected, a widely used chip compliant with EPC Gen2 technology. Its input impedance at 866 MHz is $Z_{chip}=32-j228\;\Omega$ [40], making conjugate matching with the antenna impedance essential to maximize power transfer and ensure reliable tag activation.

Although it belongs to an older generation, the Monza 3 chip remains practical for laboratory prototyping due to its quad-pad configuration (RF1, RF2, GND, GND), which allows straightforward manual soldering without specialized equipment. The Monza 3 chip exhibits a read sensitivity of approximately −15 dBm at 866 MHz, compared with about −22 dBm for the Monza R6. Despite this limitation, the experimental results obtained in this study remain satisfactory.

**Figure 4:**
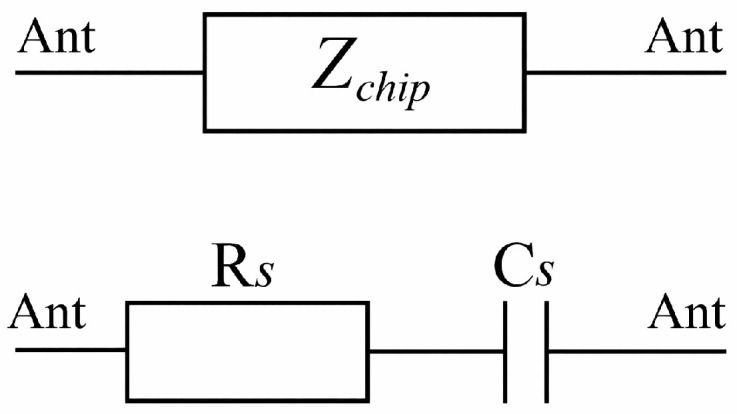
Equivalent series configuration of the Impinj Monza 3 chip used in the CST model.

For accurate impedance matching during antenna design, the chip is represented by an equivalent circuit implemented in CST Studio Suite. In this work, the chip is modeled using a series configuration, where the real part is represented by a resistance ($R_{s}$) and the reactive part by a capacitance ($C_{s}$). In the simulation environment, discrete ports define the resistive value in ohms, while lumped elements represent the capacitive component in farads, as illustrated in Figure 4.

Figure 4 illustrates the equivalent chip implementation used in CST. Although RFID chips are often described using a parallel equivalent model in the UHF RFID literature, the physically relevant quantity for impedance matching remains the complex chip impedance at the operating frequency. In the present work, the chip was implemented using an equivalent series RC representation at $f_{0}=866$ MHz, chosen so as to reproduce the same complex impedance used in the matching analysis, namely $Z_{chip}=R_{chip}-jX_{chip}$.

Accordingly, the conjugate-matching condition at the design frequency is written as

(4) $$Z_{a}\left(f_{0}\right)=Z_{chip}^{*}\left(f_{0}\right)=R_{chip}+jX_{chip}.$$

For the equivalent series implementation used in CST, the chip is represented by

(5) $$\begin{array}{cc} R_{s} & =R_{chip}, \end{array}$$

(6) $$\begin{array}{cc} C_{s} & =\frac{1}{2\pi f_{0}X_{chip}}. \end{array}$$

For completeness, the same chip can also be expressed in an equivalent parallel form at the same frequency through the admittance relation

(7) $$Y_{chip}=\frac{1}{Z_{chip}}=\frac{1}{R_{p}}+j\;2\pi f_{0}C_{p},$$

which leads to

(8) $$\begin{array}{cc} R_{p} & =\frac{R_{chip}^{2}+X_{chip}^{2}}{R_{chip}}, \end{array}$$

(9) $$\begin{array}{cc} C_{p} & =\frac{X_{chip}}{2\pi f_{0}\left(R_{chip}^{2}+X_{chip}^{2}\right)}. \end{array}$$

Therefore, the series and parallel RC representations are mathematically equivalent at the design frequency, while the matching condition remains governed by the same complex chip impedance.

## 3. Analysis of the Reference Antenna Configurations

### 3.1. Description of Antenna Configurations

**Figure 5:**
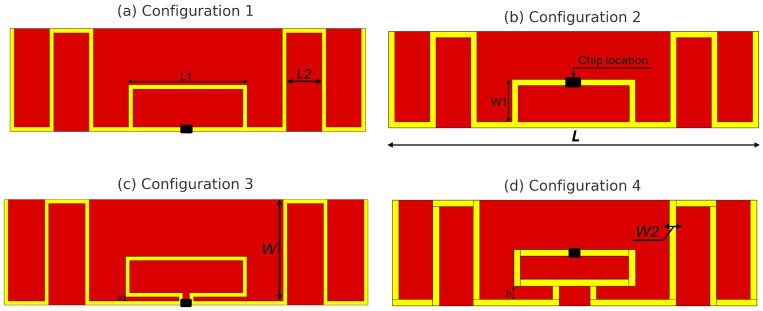
Detailed geometrical layouts of the proposed meandered UHF RFID tag for the four investigated configurations, showing the main dimensional parameters and chip locations.

The four investigated antenna configurations were defined to systematically analyze how the position of the RFID chip and the strength of the coupling-loop interaction affect the antenna–chip matching condition. The selected cases were not arbitrarily chosen; rather, they were intended to represent two main design situations. Configurations 1 and 2 correspond to direct chip mounting within the loop region, allowing the influence of chip location inside the same coupling structure to be examined. Configurations 3 and 4 introduce controlled separations between the loop and the radiating arms in order to study the progressive weakening of the electromagnetic coupling. In this way, the four layouts provide a representative set of configurations for comparing direct coupling and reduced-coupling conditions in passive UHF RFID tag design. As illustrated in Figure 5, four configurations of the Monza 3 IC placement on the meandered dipole RFID tag were investigated. The configurations differ in the IC position within the coupling-loop region and in the associated geometrical adjustments required to preserve impedance matching in the European UHF RFID band.

In Configuration 1, the IC is mounted at the central position of the loop and aligned with the lower edge of the structure. In Configuration 2, the IC is placed at the upper edge of the loop, with its position shifted vertically by $W_{1}$. In Configuration 3, the loop is separated from the dipole arms by a gap of a=1 mm, and the IC is located on the inner side of the loop. In Configuration 4, the loop geometry is further modified by introducing a separation of b=3 mm, while the IC is positioned in the upper part of the loop.

In Configurations 1 and 2, the IC is directly connected to the coupling loop, whereas Configurations 3 and 4 introduce a controlled separation between the loop and the radiating arms. This enables a comparative investigation between direct chip mounting and loop-gap integration in terms of impedance matching and tag sensitivity.

To improve the reproducibility of the proposed designs, the updated Figure 5 explicitly indicates the main geometrical parameters, including the overall antenna length *L*, antenna width *W*, loop dimensions ($L_{1}$, $W_{1}$), the secondary length parameter $L_{2}$, trace width $W_{2}$, and the separation gaps *a* and *b*. The numerical values of these parameters for the four investigated configurations are summarized in Table 1.

Modifying the IC position within the tag structure is not merely a geometrical displacement. Each configuration required specific adjustments of the antenna dimensions in order to preserve conjugate matching with the RFID chip and maintain resonance around $f_{0}=866.6$ MHz.

### 3.2. Representative Parametric Influence of the Loop Dimensions

Since the parameters $L_{1}$ and $W_{1}$ define the coupling-loop region in all four investigated configurations, they are expected to play a similar qualitative role in the impedance tuning mechanism of the proposed tag structures. To clarify this effect without introducing unnecessary repetition, a representative parametric sweep was performed on Antenna 1, while keeping all other geometrical parameters fixed.

From an impedance-matching point of view, the real part of the antenna input impedance is associated with the resistive power-transfer condition seen by the RFID chip, whereas the imaginary part governs the reactive compensation required for conjugate matching. In the present design, the loop dimensions mainly affect the reactive behavior of the structure. For this reason, the following analysis focuses on the imaginary part of the antenna impedance, which exhibits the most pronounced variation around the operating frequency.

**Figure 6:**
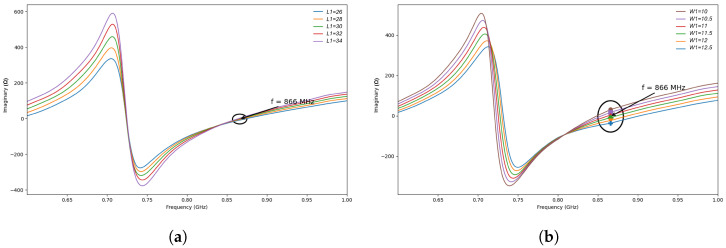
Representative parametric sweep of the loop dimensions for Antenna 1, showing the effect of $L_{1}$ and $W_{1}$ on the imaginary part of the antenna input impedance around the operating frequency. (**a**) Imaginary part of the antenna input impedance for different values of $L_{1}$. (**b**) Imaginary part of the antenna input impedance for different values of $W_{1}$.

The parameter $L_{1}$ was varied from 24 to 36 mm with a step of 2 mm, whereas $W_{1}$ was varied from 10 to 12.5 mm with a step of 0.5 mm. As shown in Figure 6, both parameters affect the impedance behavior near 866 MHz. However, the variation of $W_{1}$ produces a stronger change in the imaginary part of the antenna impedance than the variation of $L_{1}$. This behavior can be explained by the fact that $W_{1}$ directly modifies the geometry of the coupling-loop region and therefore perturbs the local reactive field distribution and the electromagnetic coupling between the loop and the radiating arms more significantly. By contrast, $L_{1}$ mainly changes the effective loop length, leading to a more gradual tuning of the impedance response.

### 3.3. Input Impedance Analysis

**Figure 7:**
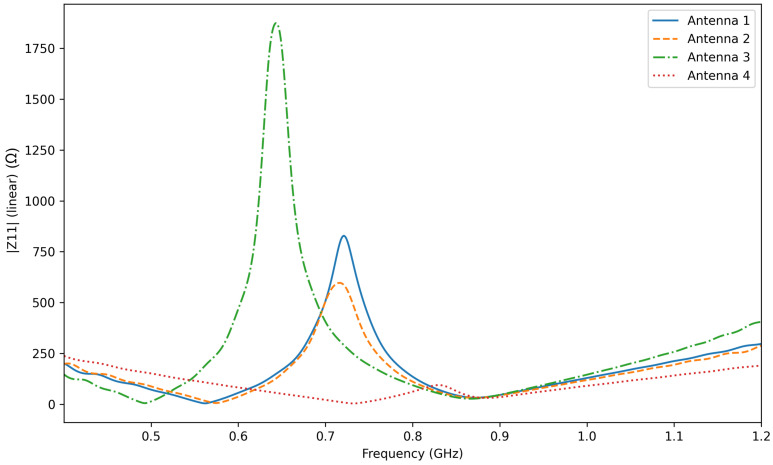
Simulated magnitude of the antenna input impedance for the four investigated UHF RFID tag configurations.

The four antenna configurations were modeled in CST Studio Suite using the Time Domain solver and optimized around the target operating frequency of $f_{0}=866.6$ MHz. A hexahedral mesh was employed, with particular attention to the thin copper traces and the fine geometrical details of the coupling-loop region in order to ensure adequate numerical accuracy. The mesh density was refined until stable impedance behavior was obtained around the operating frequency. Instead of using the reflection coefficient representation, Figure 7 presents the simulated magnitude of the antenna input impedance for the four investigated structures. This representation provides a direct comparison of how the IC location and the loop geometry affect the electrical response of the tag.

As observed in Figure 7, the different IC placements lead to noticeable variations in the impedance behavior over the considered frequency range. In particular, the impedance response around the operating frequency is strongly influenced by the coupling conditions between the loop region and the radiating arms. Configurations 1 and 2 exhibit a more favorable impedance behavior near 866.6 MHz, which indicates a better antenna–chip matching condition. By contrast, Configurations 3 and 4 show a less favorable impedance response, consistent with the introduction of the gaps a=1 mm and b=3 mm, which weaken the electromagnetic coupling between the coupling loop and the main radiating structure.

From the RFID matching point of view, the antenna performance is directly related to the power transmission coefficient between the antenna and the chip. This coefficient depends on the complex impedance relationship between the antenna and the RFID IC, and it reaches its maximum when the antenna impedance approaches the conjugate of the chip impedance. Therefore, the impedance curves shown in Figure 7 provide a direct physical interpretation of the expected power-transfer capability of each configuration: the closer the antenna input impedance is to the conjugate-matching condition at the operating frequency, the higher the achievable power transmission to the chip.

For passive UHF RFID tags, the matching condition is more appropriately interpreted through the antenna input impedance and its relationship with the complex chip impedance than through the conventional $S_{11}$ parameter alone. In this context, the power transmission coefficient between the antenna and the chip can be written as

(10) $$\tau =\frac{4R_{chip}R_{a}}{{\left|Z_{chip}+Z_{a}\right|}^{2}},$$

where $Z_{chip}=R_{chip}-jX_{chip}$ is the chip impedance and $Z_{a}=R_{a}+jX_{a}$ is the antenna input impedance. Equivalently, the complex reflection coefficient can be expressed as

(11) $$\Gamma =\frac{Z_{chip}-Z_{a}^{*}}{Z_{chip}+Z_{a}},$$

so that the transmitted power is maximized when the antenna impedance approaches the complex conjugate of the chip impedance at the operating frequency. Therefore, the impedance behavior of the proposed configurations provides a direct interpretation of the expected antenna–chip power transfer capability.

### 3.4. Fabrication of the Tag Prototypes

The proposed RFID tag prototypes were fabricated from a flexible paper-backed substrate with a total thickness of approximately 0.2 mm and a copper thickness of 0.035 mm. After the antenna geometry had been optimized in CST Studio Suite 2018, the final layout was exported in DXF format and imported into the cutting-control software associated with the Summa F1625 cutting plotter. The scale, reference, and cutting-path parameters were then adjusted before fabrication. The unwanted copper regions were subsequently removed mechanically in order to obtain the final meandered tag layouts. Therefore, the fabrication process used in this work is a cutting-and-mechanical-removal procedure rather than a chemical etching or PCB milling process. It should be noted that such a method may introduce small geometrical deviations related to cutting tolerance, alignment, or manual handling during copper removal. However, no dedicated post-fabrication dimensional metrology was carried out in the present study. This fabrication approach nevertheless enabled the practical realization of the investigated configurations under consistent material and processing conditions. It should be noted that these measurements provide a comparative experimental indication of tag activation behavior under identical test conditions, rather than a direct measurement-based validation of the simulated antenna impedance.

### 3.5. Power Sensitivity Evaluation

#### 3.5.1. Experimental Setup

While reflection coefficients provide an initial indication of impedance matching quality, the practical performance of UHF RFID tags must also be evaluated in terms of the minimum power required to activate the IC, commonly referred to as *power sensitivity*. This parameter was measured using an M6E evaluation board equipped with an RF front-end and connected via a coaxial cable to a reader antenna. The board was interfaced with a computer that controlled the measurement procedure and recorded the data.

**Figure 8:**
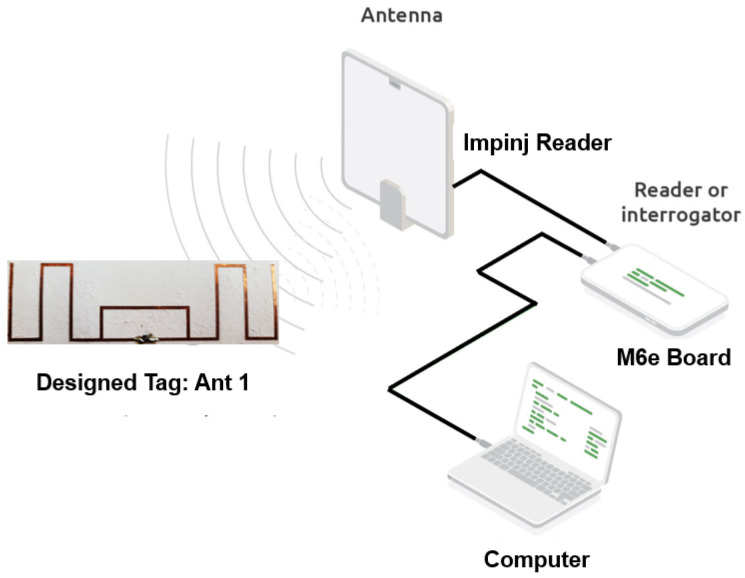
Experimental setup for sensitivity measurement of the designed RFID tags.

During testing, the fabricated tag antennas were positioned approximately 1 m from the reader antenna. The system recorded the minimum transmitted power at which each tag was successfully energized, providing a direct measure of power sensitivity, as shown in Figure 8.

#### 3.5.2. Impact of IC Placement on Tag Sensitivity

**Figure 9:**
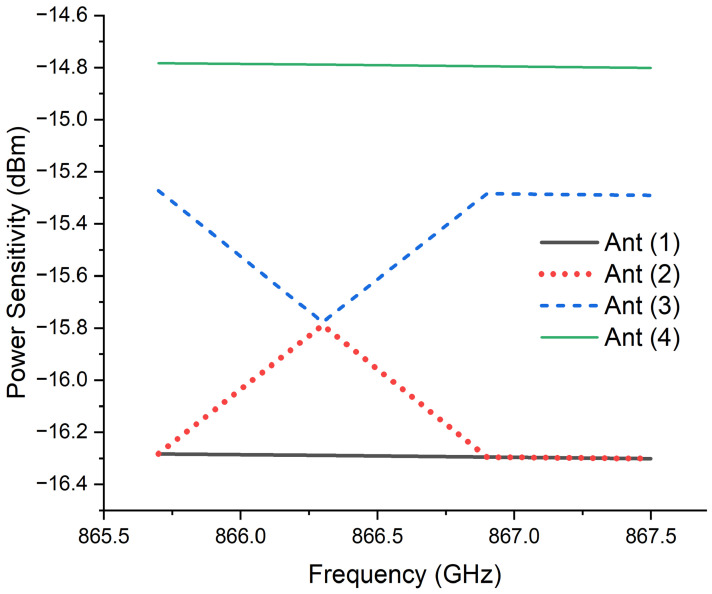
Power sensitivity of the four RFID tag antenna configurations.

The power sensitivity measurements provide a clear comparison among the four configurations, as shown in Figure 9. Antenna 1 exhibited the lowest required input power, with a minimum of −16.3 dBm at 866 MHz, confirming the advantage of placing the IC directly within the coupling loop to ensure strong conjugate impedance interaction. Antenna 2 showed nearly identical performance, with a minimum of −16.2 dBm. Antenna 3 required approximately −15.2 dBm, indicating reduced sensitivity due to partial decoupling caused by the increased loop offset. Antenna 4 demonstrated the weakest performance, with a minimum of −14.7 dBm, corresponding to a noticeable degradation in power transfer efficiency.

These results demonstrate that IC placement within the radiating structure not only affects the reflection characteristics but also directly influences tag sensitivity and the practical read range. The spatial relationship between the coupling loop and the antenna arms governs the impedance transformation efficiency, determining how effectively the IC can be energized under low-power conditions. Therefore, precise IC positioning is a critical design parameter in UHF RFID tag optimization.

## 4. Miniaturized Antenna Design and Robustness Evaluation

### 4.1. Miniaturized Antenna: Antenna 5

Based on the comparative analysis of the four reference configurations, Antenna 1 was identified as the most efficient structure and selected as the reference model for miniaturization. In this stage, additional meandered sections were introduced into the radiating arms in order to reduce the physical size while preserving the effective electrical length.

The incorporation of these folded lines initially shifted the resonance toward higher frequencies due to modifications in the current distribution. This shift was compensated through iterative adjustments of the meander lengths and trace widths, restoring resonance at 866 MHz.

**Figure 10:**
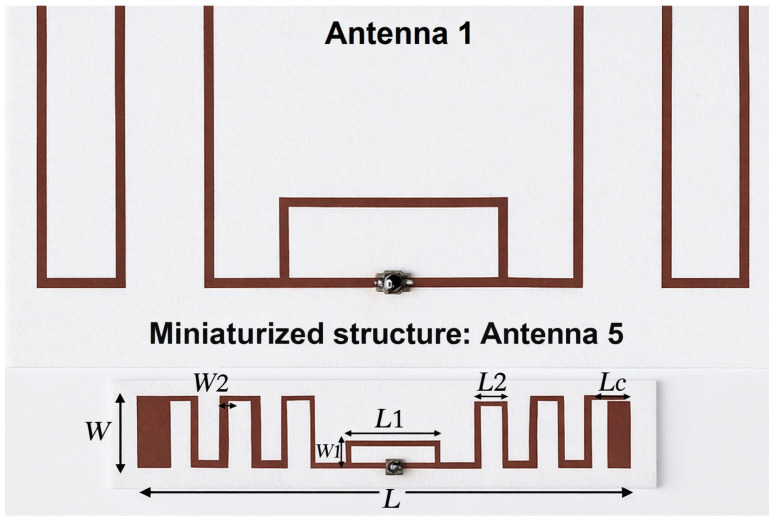
Design parameters of Antenna 1 and the miniaturized Antenna 5.

To further enhance impedance matching, a capacitive end-loading was implemented at the extremities of the structure. This technique improves reactive compensation and facilitates conjugate matching between the antenna input impedance and the RFID chip while maintaining compact dimensions. Figure 10 shows the main design parameters of Antenna 1 and the miniaturized Antenna 5.

#### Representative Parametric Optimization of Antenna 5

To clarify the miniaturization procedure of Antenna 5, a representative parametric sweep was carried out on the four main geometrical parameters that were iteratively adjusted during the final tuning stage, namely *W*, $L_{c}$, $W_{2}$, and $L_{2}$, while keeping the remaining dimensions fixed. In agreement with the impedance-based design methodology adopted throughout this work, the analysis focuses on the imaginary part of the antenna input impedance, Im(Zin), because these parameters mainly affect the reactive tuning of the miniaturized structure around the target operating frequency.

**Figure 11:**
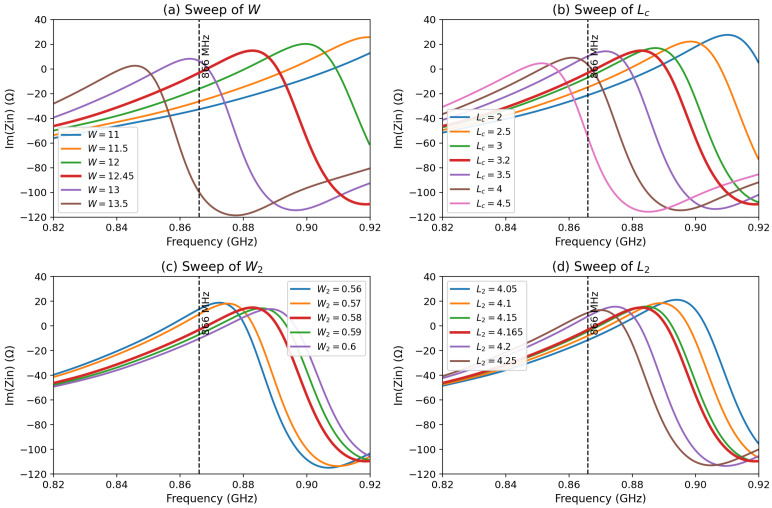
Representative parametric sweep of the miniaturized Antenna 5, showing the effect of the parameters (**a**) *W*, (**b**) $L_{c}$, (**c**) $W_{2}$, and (**d**) $L_{2}$ on the imaginary part of the antenna input impedance around the operating frequency. The dashed vertical line indicates the design frequency at 866 MHz.

Figure 11 shows the evolution of Im(Zin) for the considered parameter sweeps in the frequency range around 866 MHz. The vertical dashed line indicates the operating frequency used for the final tuning. It should be noted that a small value of Im(Zin) around this frequency indicates that the reactive part of the miniaturized structure is effectively compensated and that the antenna is tuned close to its intended operating condition. However, the exact antenna–chip conjugate-matching condition remains governed by the full complex impedance relationship, not by the imaginary part alone.

The parameter *W* controls the overall compact geometry of the radiating structure and therefore strongly affects the effective current path and the global impedance slope. As observed in Figure 11a, varying *W* produces a pronounced shift of the reactive response around the operating frequency. The selected value W=12.45 mm provides the most favorable tuning among the tested cases.

The parameter $L_{c}$ corresponds to the capacitive end-loading section introduced at the extremities of Antenna 5. Its role is mainly to provide reactive compensation and fine resonance adjustment after the meandered sections are incorporated. Figure 11b confirms that $L_{c}$ has a strong effect on the local reactive behavior near 866 MHz. The optimized value $L_{c}=3.2$ mm was retained because it provides the most suitable reactive tuning at the design frequency.

The trace width $W_{2}$ also influences the impedance response, although in a more localized manner. As shown in Figure 11c, modifying $W_{2}$ changes the current confinement and the distributed inductive–capacitive balance of the compact structure, which leads to a measurable shift of the reactive response. The value $W_{2}=0.58$ mm was selected as the best compromise.

Finally, the meander length $L_{2}$ directly affects the effective electrical length of the folded arms and therefore contributes to resonance restoration after miniaturization. Figure 11d shows that changing $L_{2}$ significantly shifts the reactive response in the vicinity of the operating frequency. The final choice $L_{2}=4.165$ mm provided the most appropriate tuning condition among the tested values.

These representative sweeps confirm that the final dimensions of Antenna 5 were not selected arbitrarily, but resulted from a controlled iterative tuning process in which *W* and $L_{2}$ mainly governed the compact resonant path, while $L_{c}$ and $W_{2}$ were used to refine the reactive compensation and improve the final impedance behavior near 866 MHz.

The optimized geometric parameters of the miniaturized antenna (Antenna 5) are summarized in Table 2. These values correspond to the final configuration obtained after integrating the meandered sections and capacitive end-loading, ensuring resonance at 866 MHz within the European UHF RFID band.

### 4.2. Frequency-Dependent Impedance and Power-Transfer Analysis

To provide a more rigorous interpretation of the antenna–chip matching behavior of the two final designs, a supplementary frequency-dependent impedance analysis was carried out for Antenna 1 and Antenna 5. In this analysis, the chip representation was removed from the excitation region, and the antenna terminal impedance was extracted from electromagnetic simulation through the excitation port. This procedure was introduced in order to separately analyze the real and imaginary parts of the antenna impedance as functions of frequency, rather than relying only on the overall impedance-magnitude representation.

**Figure 12:**
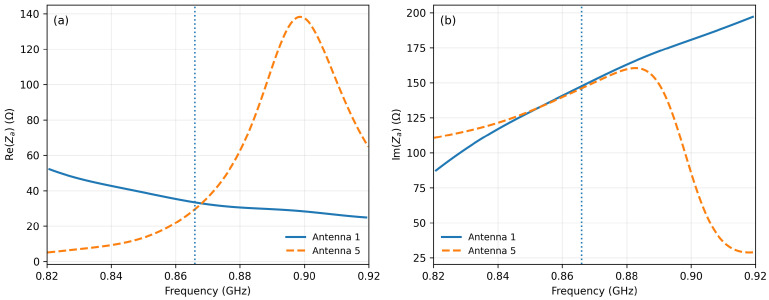
Simulated frequency-dependent antenna terminal impedance of the two final designs after removing the chip model from the excitation region: (**a**) real part of the antenna impedance for Antenna 1 and Antenna 5; (**b**) imaginary part of the antenna impedance for Antenna 1 and Antenna 5. The dashed vertical line indicates the design frequency at 866 MHz.

Figure 12a shows the real part of the simulated antenna impedance, which is associated with the resistive power-transfer behavior at the antenna terminals. Figure 12b shows the imaginary part, which reflects the reactive tuning of the structure over frequency. Presenting these two quantities separately provides a more physically meaningful interpretation of the matching behavior than the impedance magnitude alone, since conjugate matching depends on both the resistive and reactive components of the antenna impedance.

As observed in Figure 12, both Antenna 1 and Antenna 5 exhibit a clear frequency-dependent impedance behavior. The two structures differ not only in the absolute values of their real and imaginary parts, but also in the rate at which these quantities evolve around the operating frequency. This difference is consistent with the miniaturization strategy adopted for Antenna 5, where the additional meandered sections and capacitive end-loading increase current confinement and modify the reactive balance of the structure. Consequently, although the miniaturized antenna preserves a resonance behavior in the desired UHF region, its impedance evolution differs from that of the reference Antenna 1, especially in the vicinity of the tuning frequency.

It should be emphasized that the curves of Figure 12 were obtained from electromagnetic simulation and are intended to clarify the intrinsic terminal behavior of the antenna structures after removing the chip model from the excitation region. Therefore, they should not be interpreted as direct experimental impedance measurements performed with a dedicated differential fixture or probe-based setup. Their role in the present work is to improve the physical interpretation of the frequency-dependent impedance response of the two final designs within the actual scope of the study.

In order to further strengthen the interpretation of the matching behavior, the power transmission coefficient between the antenna and the chip was also evaluated as a function of frequency. For this purpose, the chip impedance was treated as frequency-dependent. The reference values provided in the manufacturer documentation for the Impinj Monza 3 chip at 866, 915, and 956 MHz were used as interpolation points, and the corresponding frequency-dependent chip impedance $Z_{chip}\left(f\right)$ was combined with the simulated antenna impedance $Z_{a}\left(f\right)$ to compute the power transmission coefficient according to

(12) $$\tau \left(f\right)=\frac{4R_{chip}\left(f\right)R_{a}\left(f\right)}{{\left|Z_{chip}\left(f\right)+Z_{a}\left(f\right)\right|}^{2}}.$$

**Figure 13:**
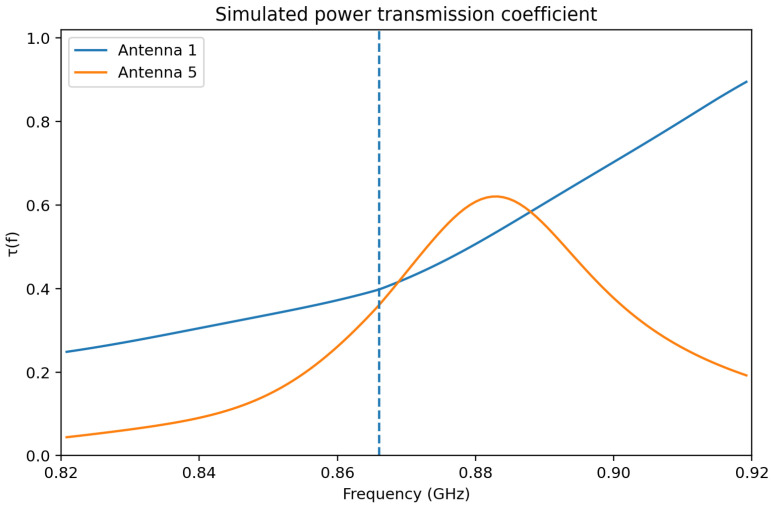
Simulated power transmission coefficient τ(f) for Antenna 1 and Antenna 5, calculated from the simulated antenna impedance and the interpolated frequency-dependent impedance of the Impinj Monza 3 chip. The dashed vertical line indicates the design frequency at 866 MHz.

Figure 13 provides a more direct evaluation of the antenna–chip matching quality than impedance inspection alone, because it combines the resistive and reactive effects of both the antenna and the chip into a single physically meaningful parameter. In this sense, τ(f) offers a clearer indication of the fraction of the available power effectively transferred from the antenna terminals to the RFID chip as the frequency varies. The results confirm that both structures are tuned to operate in the intended UHF RFID region, while also highlighting the different frequency sensitivity of the reference and miniaturized designs.

More specifically, Antenna 1 exhibits a transmission-coefficient behavior that remains more favorable around the intended operating region, which is consistent with its superior measured read-range performance and its greater robustness under material loading. By contrast, Antenna 5, although still properly tuned in free space, exhibits a more selective frequency response due to the stronger electromagnetic confinement introduced by miniaturization and capacitive end-loading. This behavior is physically consistent with the higher sensitivity of the compact structure to detuning and environmental perturbation observed in the experimental section.

It should also be clarified that the results of Figure 13 remain simulation-based matching indicators and do not constitute a direct fixture-based measurement of the antenna–chip power-transfer coefficient. Likewise, the measurements carried out with the Voyantic Tagformance Pro system are over-the-air performance measurements and not direct impedance measurements. Therefore, in the present work, the relationship between the simulated impedance-based indicators and the measured read-range behavior should be interpreted as a functional and physically motivated correlation, rather than as a strict one-to-one equivalence with direct impedance metrology. Nevertheless, the combined use of the separated real and imaginary impedance components together with the calculated τ(f) significantly improves the rigor of the matching analysis and provides a clearer justification of the observed performance differences between Antenna 1 and Antenna 5.

### 4.3. Surface Current Distribution Analysis

**Figure 14:**
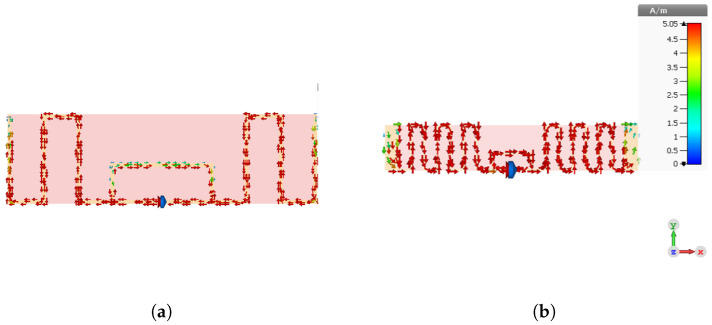
Surface current distribution comparison between Antenna 1 and the miniaturized Antenna 5 at the operating frequency of 866 MHz. (**a**) Surface current distribution of Antenna 1 at 866 MHz. (**b**) Surface current distribution of the miniaturized Antenna 5 at 866 MHz.

To provide deeper insight into the operating behavior of the proposed structures, the surface current distributions of Antenna 1 and the miniaturized Antenna 5 were analyzed at the operating frequency of 866 MHz, as shown in Figure 14. This comparison is particularly useful because the miniaturization process not only introduced additional meandered sections and capacitive end-loading but also required modifications of the loop dimensions. These geometrical changes directly affect the current path and the way in which electromagnetic energy is distributed over the radiating structure.

For Antenna 1, the current mainly flows along the external radiating arms and around the coupling-loop region. A locally weaker current-density zone can be observed along the upper horizontal section of the relatively large loop. This behavior is physically expected, since the larger loop dimensions promote current spreading over a longer path, which reduces the local current concentration in that region. In other words, the wider coupling-loop area introduces a less confined current path, leading to a moderate reduction in surface current density in the central loop section.

By contrast, the miniaturized Antenna 5 exhibits a more uniform surface current distribution along the meandered arms and around the compact loop region. This behavior indicates that the current is more effectively confined and guided through the shortened radiating path created by the meandered geometry. The stronger confinement of the current in the compact structure is consistent with the miniaturization strategy, where the effective electrical length is preserved by folding the conductor path into a smaller physical area.

It can also be observed that the current density becomes weaker in the capacitive end-loading sections located at the extremities of Antenna 5. This is also consistent with the expected electromagnetic behavior. These terminal sections mainly contribute to reactive tuning and electric-field storage rather than carrying the strongest conduction current along the main radiating path. Therefore, their role is primarily to provide capacitive compensation and resonance adjustment. This loading mechanism is associated with a more selective resonant response, which is consistent with the narrower-band behavior generally observed in compact loaded antennas.

### 4.4. Material Proximity Effects

#### 4.4.1. Measurement Setup Using Voyantic Tagformance

The performance of the fabricated tags was evaluated using the Voyantic Tagformance measurement system, a widely adopted platform for RFID characterization. The setup consists of a calibrated reader antenna and a controlled measurement station that excites the tag under test. Before performing the measurements, the Voyantic Tagformance Pro setup was calibrated using a UHF Reference Tag. All reported read-range and power-related measurements were acquired only after this calibration procedure. The reader antenna was positioned at a fixed distance of 30 cm from the fabricated tag. A polystyrene support was used to maintain a stable measurement configuration and to reduce direct environmental perturbation during the free-space characterization. The polystyrene element used in the measurement setup was introduced only as a practical low-permittivity support/spacer material to ensure mechanical stability and, in the metallic-loading case, to prevent direct electrical short-circuit between the tag and the copper surface. It was not intended to represent an ideal air-like medium in the analysis. In addition, all measurements performed with the Voyantic Tagformance Pro system were acquired only after calibration using a UHF Reference Tag.

**Figure 15:**
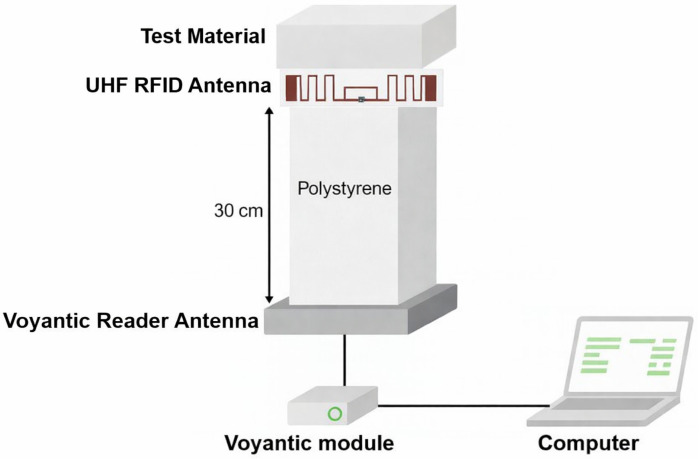
Measurement setup for the UHF RFID antenna in proximity to test materials.

The Tagformance software enables accurate extraction of key parameters, including transmitted power, backscattered power, radar cross section (RCS), theoretical read range, and orientation sensitivity across the UHF RFID band. Figure 15 illustrates the measurement setup used for the material-proximity evaluation.

#### 4.4.2. Read Range Evaluation of Antenna 1

The objective of this experiment was to evaluate tag performance in proximity to different materials representative of practical industrial environments. In the reference configuration, the antenna was placed on a polystyrene support at a fixed distance of 30 cm from the reader antenna. Under these quasi-free-space conditions, Antenna 1 achieved a maximum read range of approximately 8.6 m at 866 MHz.

When a wooden plate (20×20 cm^2^) with a thickness of 9 mm was placed above the tag, the read range decreased to approximately 5 m. Increasing the wood thickness to 15 mm further reduced the read range to about 4 m.

**Figure 16:**
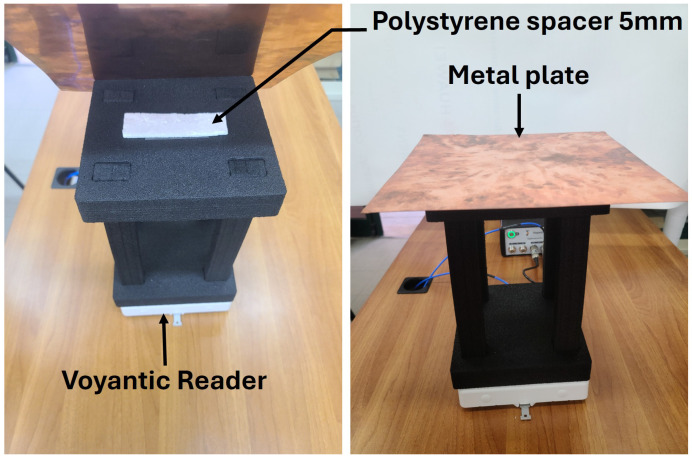
Experimental setup for RFID antenna measurement with spacer and material plate.

For metallic loading, a copper sheet was introduced. To prevent electrical short-circuiting, a 5 mm polystyrene spacer was inserted between the tag and the metallic surface, as illustrated in Figure 16.

**Figure 17:**
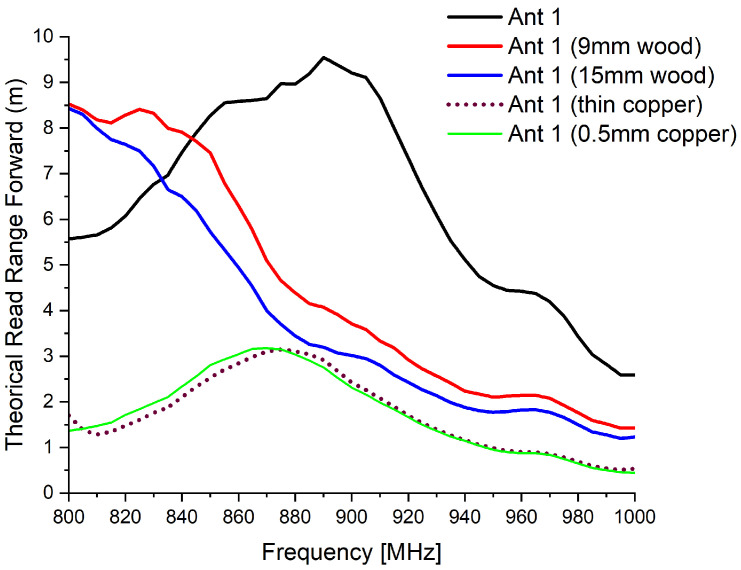
Theoretical read range of Antenna 1 under different material loading conditions.

Under metallic proximity, the antenna performance degraded further, with the read range limited to approximately 3 m. Increasing the copper thickness to 0.5 mm produced a similar effect, maintaining a read range close to 3 m at 866 MHz. Figure 17 presents the measured forward read range of Antenna 1 under various loading conditions.

#### 4.4.3. Read Range Evaluation of Antenna 5

**Figure 18:**
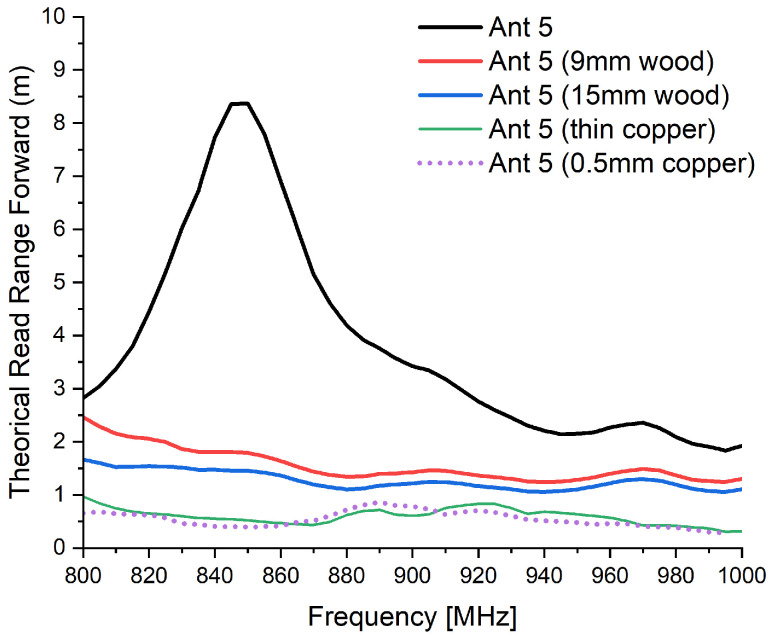
Theoretical read range of Antenna 5 under different material loading conditions.

For the miniaturized structure (Antenna 5), a noticeable reduction in performance was observed compared with Antenna 1, as shown in Figure 18. Under free-space conditions, the maximum forward read range reached approximately 6.3 m at 866 MHz.

When loaded with a 9 mm wooden plate, the read range decreased significantly to about 1.6 m and further dropped to approximately 1.3 m with a 15 mm wooden plate.

In the presence of copper layers, regardless of thickness (thin sheet or 0.5 mm), the read range was severely limited, stabilizing around 0.5 m.

This configuration was adopted to study conductive proximity effects rather than to emulate a dedicated direct-contact on-metal RFID tag architecture.

### 4.5. Commercial Tag Benchmark

To contextualize the material-proximity results obtained for the proposed antennas, additional measurements were conducted on two commercially available UHF RFID tags used as practical benchmarks. This comparison provides a reference level of read-range degradation under dielectric and conductive loading conditions.

**Figure 19:**
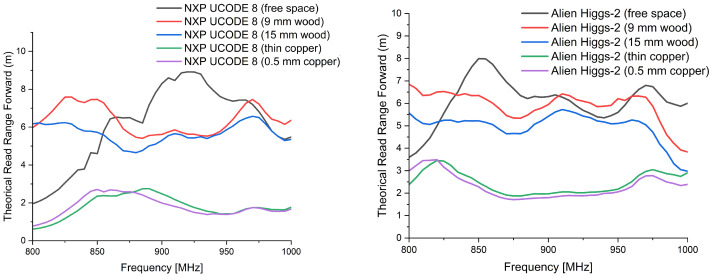
Measured read range versus frequency for two commercially available UHF RFID tags under free-space conditions and in proximity to wood and copper. (**Left**): Commercial tag B (NXP UCODE 8). (**Right**): Commercial tag A (ThinPropeller 2006, Alien Higgs-2).

Figure 19 presents the measured read range versus frequency for two commercial UHF RFID tags under three conditions: free space, wood proximity, and copper proximity.

The first benchmark, a commercial tag equipped with an NXP UCODE 8 integrated circuit, exhibits a maximum read range of approximately 6.5 m at 866.6 MHz under free-space conditions. When a wooden slab is placed in direct proximity, the read range decreases to about 5 m, corresponding to a reduction of nearly 23%. In the presence of copper, a more significant degradation is observed, with the read range reduced to approximately 2.5 m, representing a reduction exceeding 60% relative to free space.

The second benchmark, a ThinPropeller 2006 commercial tag equipped with an Alien Higgs-2 IC, achieves a free-space read range of approximately 7.2 m at 866.6 MHz. Under wood proximity, the read range decreases to about 5.5 m. In the presence of copper, a pronounced degradation occurs, limiting the maximum read range to approximately 1.9 m.

These results highlight the strong sensitivity of commercially available RFID tags to nearby materials, particularly conductive objects, despite being optimized for logistics and tracking applications.

Table 3 summarizes the measured read range values and the corresponding degradation induced by wood and copper proximity for the proposed antennas and the commercial benchmarks, under identical experimental conditions at 866.6 MHz.

#### Discussion

The material-proximity measurements were conducted to emulate realistic deployment scenarios and to evaluate the robustness of the proposed tag antennas under dielectric and conductive loading conditions. In free-space operation at 866 MHz, Antenna 1 achieves a read range of 8.6 m, comparable to the selected commercial benchmarks (7.2 m for the ThinPropeller 2006 with Alien Higgs-2 IC and 6.5 m for the UCODE 8-based tag).

Under wood loading, all tags exhibit moderate degradation, which is consistent with dielectric loading effects. The presence of a dielectric layer modifies the effective permittivity surrounding the antenna, perturbs its electrical length, and alters the antenna–IC conjugate matching condition. As a result, the available power transferred to the chip decreases, leading to a reduced read range.

A significantly stronger degradation is observed under copper proximity. Conductive loading introduces severe detuning and reduces radiation efficiency due to induced surface currents and near-field confinement. These effects increase mismatch losses and raise the minimum activation threshold of the chip. In this critical scenario, Antenna 1 maintains a read range of approximately 3 m, which is comparable to the UCODE 8 benchmark (about 2.5 m) and higher than the ThinPropeller 2006 benchmark (about 1.9 m). This confirms that the proposed reference structure remains operational and competitive under conductive loading conditions.

In contrast, Antenna 5, although compact and well matched in free space, exhibits substantially higher sensitivity to nearby conductive materials, with the read range decreasing to approximately 0.5 m under copper loading. This severe degradation shows that the miniaturized structure is not suitable, in its present form, for deployment in close proximity to conductive surfaces. The observed behavior reflects the inherent trade-off between miniaturization and environmental robustness. Because the compact geometry confines the current more strongly and stores a larger fraction of reactive energy in a reduced volume, the antenna becomes more sensitive to conductive loading and detuning effects. In addition, the reduced dimensions and closer electromagnetic interaction within the meandered structure increase the influence of the nearby metallic plane on the antenna response, leading to stronger mismatch and efficiency degradation. From a design perspective, possible mitigation strategies for the miniaturized version would include increasing the substrate or spacer thickness, introducing a larger separation from conductive surfaces, or redesigning the compact geometry using a more metal-tolerant configuration.

## 5. Conclusions

In this work, a comparative design and experimental study of passive UHF RFID tag antennas fabricated on a flexible paper-backed substrate was presented. Four reference configurations were investigated by varying the chip placement and the coupling conditions within the loop region. The results show that chip placement has a strong influence on antenna–chip matching, activation sensitivity, and read-range performance. Among the evaluated structures, Antenna 1 provided the most favorable overall behavior, achieving the best measured sensitivity and the highest read range. Based on this reference design, a miniaturized version, Antenna 5, was developed by introducing additional meandered sections and capacitive end-loading while preserving operation in the European UHF RFID band. Although the miniaturized structure remained functional in free space, it exhibited severe sensitivity to conductive loading. In particular, its strong degradation near copper shows that Antenna 5 is not suitable, in its present form, for operation close to metallic surfaces. This result highlights the trade-off between compactness and environmental robustness in aggressive RFID tag miniaturization. The comparison with commercial RFID tags further showed that Antenna 1 achieves competitive performance under both free-space and material-proximity conditions. At the same time, some limitations of the present work should be acknowledged. The dielectric loss tangent and long-term environmental stability of the paper-backed substrate were not systematically characterized, and no direct measurement of the antenna impedance or antenna-to-chip power transmission coefficient was performed. Future work should therefore focus on environmental stability analysis, repeatability assessment over multiple fabricated samples, and redesign of the miniaturized structure using more metal-tolerant solutions and newer-generation RFID chips.

## Figures and Tables

**Table 1:** Main geometrical parameters of the four investigated UHF RFID tag configurations. All dimensions are in mm.

*Parameter*	*Configuration 1*	*Configuration 2*	*Configuration 3*	*Configuration 4*
*L*	90	90	90	83
*W*	26	25	26	24
$L_{1}$	30	31.3	30	27.7
$W_{1}$	10.7	10.75	14	8.3
$L_{2}$	11.1	12	11	10.7
$W_{2}$	1.1	1.55	1	1.5
*a*	–	–	1	–
*b*	–	–	–	3

**Table 2:** Optimized design parameters of the meandered dipole antenna (Antenna 5). All dimensions are in mm.

*Description*	*Parameter*	*Value*
Antenna length	*L*	68.9
Antenna width	*W*	12.45
Loop length	$L_{1}$	11.0
Loop width	$W_{1}$	5.0
Meandering length	$L_{2}$	4.165
Capacitive loading length	$L_{c}$	3.2
Trace width	$W_{2}$	0.58
Substrate thickness	–	0.2
Copper thickness	–	0.035

**Table 3:** Comparative read range performance of the proposed tag antennas and commercially available RFID tags under different material proximity conditions at 866.6 MHz.

Tag/Antenna	IC	Free-Space (m)	Wood (m)	Copper (m)	Degradation Wood/Copper (%)
Antenna 1 (proposed)	Impinj Monza 3	8.6	4.5	3.0	47.7/65.1
Antenna 5 (proposed)	Impinj Monza 3	6.3	1.45	0.5	77.0/92.1
Commercial tag A	Alien Higgs-2	7.2	5.5	1.9	23.6/73.6
Commercial tag B	NXP UCODE 8	6.5	5.0	2.5	23.1/61.5

*Note:* Wood values for Antennas 1 and 5 correspond to average measurements within the reported intervals. Degradation percentages are calculated with respect to the free-space read range.

## Data Availability

The data presented in this study are available within the article. Additional data supporting the reported results are available from the corresponding author upon reasonable request.

## References

[1] Tariq T., Kuo W.-C., Mahmood K., Das A.K., Alenazi M.J.F. (2024). A lightweight authentication protocol for RFID-assisted supply chain management system. IEEE Internet Things J..

[2] Khan S.R., Bernassau A.L., Desmulliez M.P.Y. (2024). Passive and battery-free RFID-based wireless healthcare and medical devices: A review. IEEE J. Radio Freq. Identif..

[3] Ali W., Nizam-Uddin, Zahid M., Shoaib S. (2025). Performance analysis and design optimization of wearable RFID sensor-antenna system for healthcare applications. IEEE Access.

[4] Gentili G.B., Dori F., Iadanza E. (2010). Dual-frequency active RFID solution for tracking patients in a children’s hospital: Design method, test procedure, risk analysis, and technical solution. Proc. IEEE.

[5] Xie S., Ma C., Feng R., Xiang X., Jiang P. (2022). Wireless glucose sensing system based on dual-tag RFID technology. IEEE Sens. J..

[6] Barba A.B., Panunzio N., Amendola S., Marrocco G., Occhiuzzi C. (2025). A multi-antenna RAIN RFID sensing architecture for pharmaceutical climatic chambers. IEEE J. Radio Freq. Identif..

[7] Zhang X., Zhang X. Application of radio frequency identification technology in maintenance tracking of aircraft electromechanical equipment. Proceedings of the 2024 International Conference on Electrical Drives, Power Electronics & Engineering (EDPEE).

[8] Xiao L., Yin Y., Wu X.N., Wang J.W. (2013). A large-scale RF-based indoor localization system using low-complexity Gaussian filter and improved Bayesian inference. Radioengineering.

[9] Occhiuzzi C., Amendola S., Nappi S., D’Uva N., Marrocco G. RFID technology for Industry 4.0: Architectures and challenges. Proceedings of the 2019 IEEE International Conference on RFID Technology and Applications (RFID-TA).

[10] Komma P., Vogelbruch M., Jung M. Digital twins in industrial automation: A closer look on RFID read/write components for virtual commissioning. Proceedings of the 2024 IEEE 22nd International Conference on Industrial Informatics (INDIN).

[11] Othmani H., Beldi S., Azizi M.K. Design of an SHF RFID reader antenna for access control and security systems. Proceedings of the 2024 IEEE International Conference on Advanced Systems and Emergent Technologies (IC_ASET).

[12] Nappi S., Amendola S., Ramacciotti M., Zambonini E., D’Uva N., Camera F., Miozzi C., Occhiuzzi C., Marrocco G. UHF RFID system for the predictive maintenance of a filter press: A real use case. Proceedings of the 6th International Conference on Smart and Sustainable Technologies (SpliTech).

[13] Shimizu K., Wang S., Kai H., Takahashi H., Shimizu A. A lightweight and secure one-time RFID authentication protocol based on SAS-L2. Proceedings of the 2024 IEEE International Conference on Consumer Electronics-Asia (ICCE-Asia).

[14] Panda J.R., Saladi A.S.R., Kshetrimayum R.S. (2011). A compact printed monopole antenna for dual-band RFID and WLAN applications. Radioengineering.

[15] Riaz M.A., Abdullah Y., Shahid H., Amin Y., Akram A., Tenhunen H. (2018). Novel butterfly slot based chipless RFID tag. Radioengineering.

[16] Chaffai K.M., Berenguer R., Rezola A., Diaz J., Solar H., Beriain A. Exploring dual-frequency implementation for semi-passive RFID tags. Proceedings of the 39th Conference on Design of Circuits and Integrated Systems (DCIS).

[17] Nath B., Reynolds F., Want R. (2006). RFID technology and applications. IEEE Pervasive Comput..

[18] Siakavara K., Goudos S., Theopoulos A., Sahalos J.N. (2017). Passive UHF RFID tags with specific printed antennas for dielectric and metallic objects applications. Radioengineering.

[19] Marrocco G. (2008). The art of UHF RFID antenna design: Impedance-matching and size-reduction techniques. IEEE Antennas Propag. Mag..

[20] Räsänen M., Holopainen J., Bergman J., Kuosmanen M., Viikari V. Small on-metal passive UHF RFID transponders with long read ranges. Proceedings of the 18th European Conference on Antennas and Propagation (EuCAP).

[21] Bouazza H., Lazaro A., Bouya M., Hadjoudja A. (2020). A planar dual-band UHF RFID tag for metallic items. Radioengineering.

[22] Nikitin P.V., Rao K.V.S., Lam S.F., Pillai V., Martinez R., Heinrich H. (2005). Power reflection coefficient analysis for complex impedances in RFID tag design. IEEE Trans. Microw. Theory Tech..

[23] Chen R., Yang S., Penty R.V., Crisp M. UHF RFID reader sensitivity requirements due to poor tag matching. Proceedings of the 12th IEEE International Conference on RFID Technology and Applications (RFID-TA).

[24] Dobrykh D., Yusupov I., Slobozhanyuk A., Filonov D., Ginzburg P. Compact long-range ceramic RFID tag for on-metal and non-metal applications. Proceedings of the 12th IEEE International Conference on RFID Technology and Applications (RFID-TA).

[25] Tan J.-I., Lee Y.-H., Lim E.-H. Design of a compact on-metal RFID tag with a pair of planar inverted-L antennas (PILAs). Proceedings of the 12th IEEE International Conference on RFID Technology and Applications (RFID-TA).

[26] Franchina V., Michel A., Nepa P., Salvatore A. Compact in-metal UHF RFID tag for manufactured metallic components. Proceedings of the 3rd International Conference on Smart and Sustainable Technologies (SpliTech).

[27] Nabavi S., Anabestani H., Bhadra S. A printed paper-based RFID tag for wireless humidity sensing. Proceedings of the IEEE Sensors Conference.

[28] Wang Y., Liu J., Xie L., Wen G. (2013). An ultra-low-power oscillator with temperature and process compensation for UHF RFID transponder. Radioengineering.

[29] Kanjilal R., Kucuk M.F., Uysal I. (2025). Human activity recognition: A review of RFID and wearable sensor technologies powered by AI. IEEE J. Radio Freq. Identif..

[30] Renuka N., Chin N.N., Ismail W. Embedded RFID tracking system for hospital application using WSN platform. Proceedings of the IEEE International Conference on RFID Technologies and Applications (RFID-TA).

[31] Franchina V., Ria A., Michel A., Bruschi P., Neppa P. A compact UHF RFID ceramic tag for high-temperature applications. Proceedings of the IEEE International Conference on RFID Technology and Applications (RFID-TA).

[32] Albrecht J., Dudek R., Auersperg J., Pantou R., Rzepka S. Thermal and mechanical behaviour of an RFID based smart system embedded in a transmission belt determined by FEM simulations for Industry 4.0 applications. Proceedings of the 16th International Conference on Thermal, Mechanical and Multi-Physics Simulation and Experiments in Microelectronics and Microsystems.

[33] Cummins D., Claucherty E., Nowaczyk J., Patel N., Aliakbarian B. A practical assessment of integrating RFID into over-the-counter liquid suspension supply chains. Proceedings of the IEEE International Conference on RFID Technology and Applications (RFID-TA).

[34] Yeh C.-H., Ho P.-S., Lin C.-W., Sim C.-Y.-D. Circularly polarized UHF RFID tag antenna with capacitive loading technique. Proceedings of the 5th IEEE Asia-Pacific Conference on Antennas and Propagation (APCAP).

[35] Loo C.-H., Elmahgoub K., Yang F., Elsherbeni A., Kajfez D., Kishk A., Elsherbeni T., Ukkonen L., Sydanheimo L., Kivikoski M. (2008). Chip impedance matching for UHF RFID tag antenna design. Prog. Electromagn. Res..

[36] Ripin N., Rahim M.K.A., Lim E.-H., Dewan R., Murad N.A. Metal-tolerant UHF RFID tag antenna with a comb-shaped design. Proceedings of the IEEE Asia-Pacific Conference on Applied Electromagnetics (APACE).

[37] El Ahmar L., Errkik A., Zbitou J., Oukaira A., Bouzida I., Talbi L., Lakhssassi A. (2024). A compact meander flexible UHF tag for industrial applications. e-Prime Adv. Electr. Eng. Electron. Energy.

[38] Gallo M., Viola A., Romagnoli G. (2025). The Contribution of RFID to Circular Economy Deployment: A Literature Review. Int. J. RF Technol. Res. Appl..

[39] Wang X., Kuznetcov M., Jiang W., Tang Z., Wei Z., Zhang A., Wei N., Li X. (2025). Printed RFID systems for sustainable IoT: Synergistic advances in conductive inks, antenna architectures, and scalable manufacturing. Front. Electron..

[40] Impinj, Inc. (2010). Monza^®^ 3 Tag Chip Datasheet, IPJ-W1002-A00, IPJ-W1002-C00.

